# Supplementary material for: Genome-Wide Identification and Characterization of the TIFY Gene Family and Their Expression Patterns in Response to MeJA and Aluminum Stress in Centipedegrass (Eremochloa ophiuroides)
Source: Plants (Basel). 2024 Feb 5;13(3):462. doi: 10.3390/plants13030462 (PMC10857321; doi:10.3390/plants13030462)
Supplement: Supplementary file 1 [file plants-13-00462-s001.zip › Figure S2.pdf]

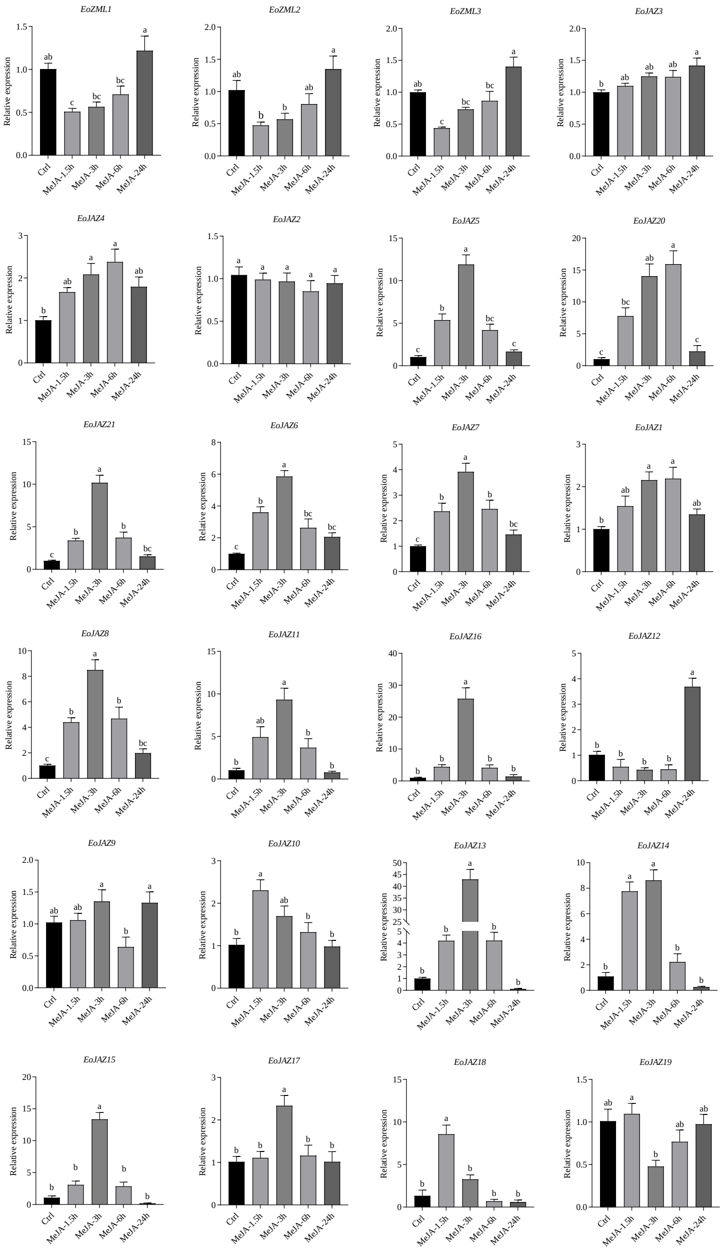

Figure S2: The results of qRT-PCR analysis for *EoTIFY* family genes in response to MeJA. Means with different letters (a, b, c) are significantly different between treatments by the Tukey's HSD test ($p < 0.05$).
